# Neoplastic and Autoimmune Comorbidities in Patients with Primary Cutaneous B-Cell Lymphoma

**DOI:** 10.3390/hematolrep15010016

**Published:** 2023-02-27

**Authors:** Roberto Mazzetto, Jacopo Tartaglia, Alvise Sernicola, Mauro Alaibac

**Affiliations:** Dermatology Unit, Department of Medicine (DIMED), University of Padua, 35121 Padova, Italy

**Keywords:** primary cutaneous B-cell lymphoma, non-Hodgkin lymphoma, cancer, autoimmunity, comorbidity

## Abstract

Primary cutaneous B-cell lymphomas (PCBCLs) constitute a rare subset of non-Hodgkin lymphoma (NHL), with distinctive clinical and biological characteristics. The risk of autoimmune or neoplastic comorbidities in subjects with NHL has been extensively reported in the literature, but the data available are not directly applicable to PCBCLs. The aim of our study was to determine the frequency of relevant medical conditions, with a primary focus on autoimmune and neoplastic disorders, in subjects with PCBCL. We performed a retrospective observational study involving 56 patients diagnosed histologically with PCBCL and 54 sex- and age-matched controls. Our results show a statistically significant association for neoplastic comorbidities in general (41.1% vs. 22.2%, *p* = 0.034) and hematological malignancies specifically (19.6% vs. 1.9%, *p* = 0.0041) with PCBCL compared to controls. We did not highlight a statistically significant difference in the frequency of autoimmune comorbidities (21.4% vs. 9.3%, *p* = 0.1128) and of chronic viral hepatitis (7.1% vs. 0, *p* = 0.1184). Finally, type 2 diabetes (19.6% vs. 1.9%, *p* = 0.0041) was significantly associated with PCBCL. Our preliminary data supporting the association between PCBCLs and neoplastic disorders suggest that altered immune surveillance may be a common predisposing mechanism.

## 1. Introduction

Despite the abundance of data in the literature regarding the association between non-Hodgkin lymphoma (NHL) and autoimmune or neoplastic comorbidities, there is a lack of studies evaluating the association between these comorbidities and primary cutaneous B-cell lymphoma (PCBCL). This subgroup has distinct biological and clinical characteristics compared to the nodal types of non-Hodgkin lymphoma and is generally associated with a more indolent course [1]. Specifically, the PCBCLs include three major entities: primary cutaneous follicular lymphoma, primary cutaneous lymphoma of the marginal zone and primary diffuse cutaneous large B-cell lymphoma, leg type [2]. Primary cutaneous follicular lymphoma and primary cutaneous lymphoma of the marginal zone are considered particularly indolent and are associated with a disease-specific 5-year survival greater than 95% [3]. By contrast, primary cutaneous diffuse large B-cell lymphoma is characterized by biologically aggressive behavior, a higher frequency of extra-nodal involvement and a significantly worse prognosis, with a 5-year disease-specific survival of 50% [2]. Since these differences set PCBCLs significantly apart from other subtypes of NHL, the disease associations established in the literature for extra-cutaneous NHL may not apply to PCBCLs. In this regard, there is a lack of published evidence in the literature concerning the comorbidities associated with PCBCLs. Some data were provided by Guitart et al., who investigated possible associations between PCBCLs and other relevant medical conditions in a cohort of 80 patients, highlighting a high incidence of gastrointestinal diseases (a broad category of conditions including gastroesophageal reflux, gastric ulcer, irritable bowel syndrome and inflammatory bowel disease) [4]. Moreover, the authors reported a significantly increased incidence of autoimmune diseases, such as Hashimoto’s thyroiditis, systemic lupus erythematosus (SLE) and Sjogren’s syndrome. Finally, a history of extracutaneous malignant tumors was found to be more frequent in patients with cutaneous marginal zone B-cell lymphomas (*p* = 0.05).

Recently, S. Hu et al. evaluated the association between NHL and autoimmune comorbidities in a large cohort of patients [5]. In total, 2.9% of the patients were affected by autoimmune comorbidities and the diseases most commonly associated with NHL were Sjogren’s syndrome, autoimmune cytopenia, rheumatoid arthritis, SLE, Hashimoto’s thyroiditis, and dermatomyositis/polymyositis. Other studies evaluated these associations. For example, M. Fallah et al. studied a cohort of 878,161 patients with autoimmune diseases to determine whether there was an increased risk of NHL in this patient group [6]. A significant increase in the risk of NHL in patients with autoimmune comorbidities was found in both sexes. The risk of all the most common histological subtypes of NHL was found to be significantly higher in patients with autoimmune diseases. Although subtype analysis highlighted an increased risk for cutaneous/peripheral T-cell and anaplastic T-cell lymphomas, the risk for PCBCLs was not assessed individually. In physiopathological terms, the causes of NHL remain poorly understood. Nonetheless, the association with autoimmune diseases shown by several large studies in the literature strongly supports their role as important predisposing factors [7,8]. First, the presence of continuous antigenic pressure and chronic inflammation in patients with autoimmune conditions stimulates the proliferation of T and B lymphocytes, which leads to an increased risk of the accumulation of genetic mutations in these cells, which in turn is associated with lymphomagenesis [9]. Second, the chronic use of immunosuppressive drugs, whose role in promoting oncogenesis is well-known, in patients with autoimmune diseases could be an additional predisposing factor [10].

Finally, the literature shows an increased risk of neoplastic comorbidities, with Hodgkin’s lymphoma, lung cancer, brain cancer, melanoma, and non-melanoma skin cancer being the most common in patients with NHL [11,12,13,14]. 

The aim of our study is to assess whether PCBCL patients are at risk for other relevant medical conditions, with a primary focus on autoimmune and neoplastic disorders.

## 2. Materials and Methods

### 2.1. Study Design and Setting

We performed a retrospective observational study employing the Galileo e-health application (Dedalus Italia S.p.A., Florence, Italy), collecting our hospital’s electronic medical records. This platform registers sociodemographic data, as well as diagnoses and written reports generated during outpatient visits and hospitalization. Electronic medical records were reviewed to collect data for comorbidities in patients diagnosed histologically with PCBCL from inception to 13 April 2022. 

Our study included 56 patients with a histological diagnosis of PCBCL according to the 2018 update of the WHO-EORTC classification for primary cutaneous lymphomas [14]. Specifically, 37 of the patients had primary cutaneous follicle center lymphoma, 13 primary cutaneous marginal zone lymphomas, and 6 had primary cutaneous diffuse large B-cell lymphoma. Our patient population consisted of 32 females and 24 males with a median age of 66 years. Subjects attending our outpatient office for routine skin examination were included in a group of 54 sex- and age-matched healthy controls. The control group was chosen from individuals who visited our department, either voluntarily or in response to awareness campaigns, for a general dermatologic assessment and without a specific request for a suspicious skin lesion to be evaluated. Health data were retrospectively collected from our hospital’s electronic medical records. Demographic characteristics are reported in Table 1. 

In both the PCBCL and the control groups, additional diagnoses were systematically investigated demonstrating the following comorbidities: thyroiditis, systemic lupus erythematosus, Behcet’s disease, lung cancer, monoclonal gammopathy of undetermined significance (MGUS), breast cancer, chronic lymphocytic leukemia/lymphoma, thyroid cancer, essential thrombocythemia, prostate cancer, colon cancer, melanoma, basal cell carcinoma, gastroenteritis, gastroesophageal reflux disease, heart transplant, HIV, hypogammaglobulinemia, HBC, HCV, type 2 diabetes (DM2), ulcerative colitis, and psoriasis. To mitigate the confounding effect of age, we compared two samples with similar ages considering that the assessed comorbidities are more prevalent among the elderly: mean age ± standard deviation was 68.7 ± 15.4 years in the PCBCL group and 66.8 ± 7.99 years in the control group (Table 1).

### 2.2. Ethics

Ethical review and approval were waived for this study, as they were not required by our Institutional Ethics Committee considering the retrospective nature of this observational registry study. Our hospital’s electronic medical records database was used in compliance with relevant legislation regarding data protection and patient privacy and the study was performed according to the principles of good clinical practice. Informed consent was obtained from all subjects involved in the study.

### 2.3. Statistical Analysis

Descriptive statistics were expressed as median and mean values or as absolute and relative frequencies (percentages). The chi-squared test was performed to compare frequencies of comorbidities between patients and controls. Fisher’s exact test was also used for analysis in cases in which frequency was below 10. Statistical significance was set for a value of *p* < 0.05. Statistical analyses were performed using Microsoft Excel (Microsoft Corporation, Redmond, WA, USA).

## 3. Results

### 3.1. Malignant Neoplasms

A history of systemic malignant neoplasms was observed in twenty-three of the patients (41.1% vs. 22.2%, *p* = 0.034, chi-squared test), eleven of whom (19.6%) had a hematological disorder, including MGUS, chronic lymphocytic leukemia or other non-cutaneous lymphomas and essential thrombocythemia. Therefore, we found a statistically significant association between PCBCL and hematological diseases (19.6% vs. 1.9%, *p* = 0.0041, Fisher’s exact test). The MGUS had a higher frequency in the PCBCL than in the controls (12.5% vs. 1.9%); however, this difference did not reach statistical significance (*p* = 0.0608, Fisher’s exact test; Table 2).

To further investigate this aspect, we obtained available data on the immunoglobulin phenotypes of MGUS and PCBCL in our patients, which are presented in Table 3. We are unable to provide the immunoglobulin heavy-chain profile of the cutaneous lymphoma clone in all the MGUS patients, since this assessment was not performed by our laboratory; however, the light-chain expression of PCBCL is reported when present.

Finally, the diagnosis of basal cell cancer was considered separately from the other neoplastic diseases in our analysis in consideration of the high frequency of this condition in the age groups represented in our study population: this type of skin tumor was diagnosed in 7 (12.5%) of the subjects with PCBCL and in 10 (18.5%) of the controls, showing no statistically significant difference between the two groups (*p* = 0.4367, Fisher’s exact test).

### 3.2. Autoimmune Diseases

Furthermore, our study showed no statistically relevant association between autoimmune diseases and PCBCL. We reported autoimmune thyroiditis as the most common pathology both in patients and controls, while SLE and Behcet’s disease were observed only in the PCBCL cohort group; in any case, none of these comorbidities showed a statistical association with PCBCL (21.4% vs. 9.3%, *p* = 0.1128, Fisher’s exact test; Table 4).

### 3.3. Other Comorbidities

Even though three patients (5.4%) suffered from a form of immunosuppression (hypogammaglobulinemia, HIV and heart transplant) while none of the controls did, we did not find a statistically significant association with an immune disorder (*p* = 0.2434, Fisher’s exact test). Surprisingly, we found eleven patients with DM2 whose results were significantly associated with PCBCL (19.6% vs. 1.9%, *p* = 0.0041, Fisher’s exact test).

Moreover, 7.1% of the cases had chronic viral hepatitis (HBV or HCV), which was found in none of the controls, although a statistical association was not reached (*p* = 0.1184, Fisher’s exact test). 

Finally, eleven controls (20.4%) had a history of gastrointestinal tract disorders (gastroenteritis, GERD, ulcerative colitis) while just eight (14.3%) among patients with PCBCL reported this (*p* = 0.4558, Fisher’s exact test). Other notable comorbidities, such as psoriasis, did not reach statistical significance. Table 5 summarizes the results for the other conditions not included in the previous groups.

## 4. Discussion

Our data suggest an association between neoplastic comorbidities and hematological disorders with PCBCL. The relationship between NHL and other oncological comorbidities is well documented in the literature, with several published studies reporting this association. In particular, Chattopadhyay et al. investigated the risk of second primary cancers (SPC) in NHL patients by conducting a bidirectional analysis (that is, of second primary cancers after NHL and NHL as SPC) [15]. This analysis was performed using data from a large cohort of 19,833 patients. After NHL diagnosis, there was an increased risk of Hodgkin’s lymphoma, squamous cell carcinoma, kidney cancer, melanoma, bladder cancer, colorectal cancer, and upper aerodigestive tract cancer. The highest relative risk was found with hematological SPCs (primarily Hodgkin’s lymphoma).

Our data show a statistically significant association between PCBCL and neoplastic comorbidities in general and hematological neoplasms specifically. The mechanism underlying this association is not known, but several hypotheses have been formulated. Radiotherapy and chemotherapy, which are widely used treatments for NHL, may be the underlying causes of many SPCs in NHL patients, as their mutagenic and oncogenic potential is a well-known side effect [16]. However, this hypothesis alone may not be sufficient to explain the increased incidence of neoplastic comorbidities in PCBCL patients. As a matter of fact, given their particularly indolent course, PCBCLs are rarely treated with the chemo- and radiotherapy approaches often used for extracutaneous NHL. One of the hypotheses proposed to explain this association is the presence of individual immune-system dysfunction in patients with NHL [17], regardless of iatrogenic immune suppression. It has been proposed that this immune imbalance may depend on the down-regulation of T-cell function driven by NF-kB, which contributes to immune suppression by inducing the activation of immune-suppressor cells [18].

Among the hematological disorders, the 12.5% frequency of MGUS in PCBCL, although not statistically significant, may hint at an altered immune surveillance, which might favor PCBCL onset. Furthermore, it may also suggest a hypothetical pathological mechanism in common. In this regard, a similar association was observed in Waldenstrom macroglobulinemia (WM) patients by Varettoni et al. who found a higher risk of second cancers compared with the general population [19]. On the other hand, monoclonal gammopathies might also be the result of immunoglobulin production by PCBCL and, in this case, would be regarded as serological markers. A retrospective study conducted on 23 patients with PCMZL demonstrated concordant paraproteins in tissue samples and in the blood, suggesting that in PCMZL paraproteinemia may serve as a tumor marker [20].

In our study, there was no shared light-chain expression between MGUS and PCBCL, considering cases for which these data were available. According to these observations, we hypothesized that MGUS might be a comorbidity rather than just a serological marker of PCBCL. However, we were not able to demonstrate a significant association between MGUS and PCBCL in our sample and further data will be required to support this hypothesis. 

We found a statistically significant association between DM2 and PCBCLs. This association could also indicate the presence of a “dys-immune” condition in PCBCL patients. The DM2 was diagnosed before the discovery of PCBCL in our patient group: this temporal association allowed us to rule out the possibility that DM2 could be a side effect of subsequent steroid therapy administered for the treatment of PCBCL. Although it is well known that DM2 and metabolic syndrome are risk factors for many neoplastic disorders [21], we are the first to report a significantly higher prevalence of DM2 in PCBCL patients. Hyperglycemia in diabetes is thought to cause dysfunction in the immune system through a wide range of mechanisms, which include impaired cytokine production, the inhibition of leukocyte recruitment, dysfunction in natural killer cells and the inhibition of antibodies and complement function [22]. All these mechanisms could contribute to a reduction in the effectiveness of tumor surveillance and, ultimately, to an increase in carcinogenesis. Another mechanism through which diabetes is hypothesized to promote carcinogenesis is the insulin-like growth factor 1 (IGF-1)-mediated stimulation of cell proliferation. Since IGF-1 receptors have been found in a variety of human malignancies, insulin may have an influence on cancer cell proliferation in vivo. Insulin stimulates the synthesis of IGF-1 in the liver by up-regulating growth hormone receptors. Hyperinsulinemia can also raise IGF-1 levels by lowering IGF-binding-protein production in the liver [23].

Moreover, our data show hepatotropic virus infection (HBV and HCV) in 7.1% of the subjects with PCBCLs and in none of the controls, although this difference did not reach statistical significance. The association between chronic hepatotropic virus infection and extrahepatic carcinogenesis (most notably NHL) is well known in the literature [24]. The very high prevalence of HCV infection in individuals with mixed cryoglobulinemia was the original observation that prompted the further exploration of this link [25]. The relative risk (RR) of all forms of NHL among HCV-positive patients was determined to be 2.5 in a meta-analysis that included 15 studies [26]. The link between NHL and HBV has received far less attention than the link between NHL and HCV. Nonetheless, a meta-analysis taking into account 12 studies confirmed the presence of a statistically significant association between chronic HBV infection and NHL [27]. Although the exact mechanism underlying this association is not known, several hypotheses have been formulated. The first proposed mechanism is chronic antigenic stimulation: when B cells undergo chronic, antigen-driven proliferation, there is the possibility of these cells accumulating mutations in their DNA, leading to the emergence of a malignant B-cell clone [28]. Another proposed mechanism is the direct viral infection of B lymphocytes: HCV-infected cells, including B cells, have been found to have a mutator phenotype due to the induction of activation-induced cytidine deaminase and the production of error-prone DNA polymerase [29]. While each of these mechanisms could account for an increased frequency of PCBCLs in patients affected by chronic hepatitis, our results did not demonstrate a statistically significant correlation between the two conditions, and additional data will be necessary to support this potential association. 

Further in-depth research is required to better understand how these comorbidities are related to PCBCL and how they may affect the prognoses of these individuals. While we were not able to assess the influence of all the comorbidities on disease, we obtained specific data on the immunoglobulin phenotypes in a subset of patients with PCBCL and MGUS, which are presented in Table 3. Although immunosuppression is a known risk factor in lymphoproliferative disorders, this study highlights how patients with PCBCL are not affected by conditions that down-regulate the immune system, such as organ transplant or HIV infection. Due to the rarity of PCBCL, our investigation did not deal with the different lymphoma subtypes separately, grouping together entities with indolent as well as aggressive behavior. In this regard, it has been reported that aggressive forms of CBCL are generally observed in the context of immunosuppression [30,31]. 

Contrary to reports by Guitart et al., we did not find a statistically significant association with autoimmune diseases [4]. From the findings in the literature, it is known that the chronic inflammatory stimulus caused by autoimmune diseases increases the risk of NHL [31]. The lack of a statistically significant association between autoimmune conditions and PCBCLs in our patient cohort most likely depends on the small size of our sample. In contrast to Guitart et al.’s observations, we did not find a statistically significant increase in gastrointestinal tract disorders in patients with PCBCL, hinting at probable mechanisms related to PCMZL pathogenesis that are not shared by the other subtypes of PCBCL included in our cohort. 

## 5. Conclusions

In conclusion, our data show a statistically significant increase in the frequency of neoplastic comorbidities in patients with PCBCL compared to controls. More specifically, we found a significant association between PCBCLs and hematological disease. By contrast, autoimmune diseases did not show a statistical association. Moreover, DM2 was significantly associated with PCBCL. The immunological events induced by these conditions are responsible for different types of immune dysregulation, suggesting that altered immune surveillance might favor PCBCL onset. The main limitation of our study was the insufficient number of subjects in the PCBCL cohort and the healthy control cohort, which prevented further robust conclusions and an additional analysis of the reciprocal associations between comorbidities based on this set of data. While we were unable to include more patients due to the rarity of this condition, our sample size is consistent with that of other studies in the published literature on PCBCL [4,20]. We hope to be able to contribute to future multicenter studies that can collect robust data to support our preliminary findings on the risk factors and comorbidities in patients with PCBCL. A further limitation of the present study was that our sample of healthy subjects was not well suited to the controls, constituting a quite heterogenous population compared with that of the subjects with PCBCL. Specifically, our age- and sex-matched controls showed a relatively high rate of malignancy, but their characteristics were similar to those of healthy controls included in previous studies [4]. To mitigate the risk of bias, an additional control group should be enrolled to provide a validation cohort for future studies. Moreover, the possibility of using patients with systemic DLBCL also presenting with skin lesions as a control group to overcome these limitations was not feasible for the present study due to the inadequate number of controls with these characteristics. Finally, future studies comparing subtypes of PCBCL with their systemic counterparts are needed to achieve an in-depth understanding of the clinical behavior and disease associations of this rare group of cutaneous lymphoproliferative disorders.

## Figures and Tables

**Table 1:** Demographic characteristics of cases and control populations.

		PCBCL	Controls
Number of participants		56	54
Sex	Males	24	23
	Females	32	31
Age, years	Mean (SD)	68.7 (15.4)	66.8 (7.99)
	Median	66	66.5

Abbreviations: PCBCL, primary cutaneous B-cell lymphoma; SD, standard deviation.

**Table 2:** Frequency of neoplastic diseases in patients with PCBCL and controls.

Comorbidities	PCBCL		Controls		*p* Value
Neoplastic diseases	number	%	number	%	
Lung cancer	1	1.8	1	1.9	
MGUS	7	12.5	1	1.9	
Breast cancer	3	5.4	4	7.4	
CLL/Other non-cutaneous lymphomas	2	3.6	0	0	
Thyroid cancer	1	1.8	1	1.9	
ET	2	3.6	0	0	
Prostatic cancer	1	1.8	2	3.7	
CRC	3	5.4	1	1.9	
Melanoma	3	5.4	1	1.9	
Bladder cancer	0	0	1	1.9	
Total	23	41.1	12	22.2	0.034 *
Basal cell carcinoma	7	12.5	10	18.5	0.4367

* Statistically significant values are reported and marked with *. Abbreviations: CLL, chronic lymphocytic leukemia; CRC, colorectal cancer; ET, essential thrombocythemia; MGUS, monoclonal gammopathy of undefined significance; PCBCL, primary cutaneous B-cell lymphoma.

**Table 3:** Immunophenotype and histological correlations in subjects with MGUS and PCBCL.

	MGUS		PCBCL	
	Type of monoclonal immunoglobulin	Bence-Jones protein	Histological subtype	Immunoglobulin light-chain
1	IgM and Kappa light chains	Detected	PDLBCL	Not expressed
2	IgG and Kappa light chains	Not detected	PCMZBCL	Lambda
3	IgG and Kappa light chains	Detected	PCFCL	Not expressed
4	IgG and Lambda light chains	Not detected	PCMZBCL	Kappa
5	IgM and Lambda light chains	Not detected	PCMZBCL	Not expressed
6	IgM and Lambda light chains	Not detected	PCFCL	Not expressed
7	IgG and Lambda light chains	Not detected	PCFCL	Not expressed

Abbreviations: MGUS, monoclonal gammopathy of undetermined significance; PCBCL, primary cutaneous B-cell lymphoma; PCFCL, primary cutaneous follicle center lymphoma; PCMZBCL, primary cutaneous marginal zone B-cell lymphoma; PDLBCL, primary diffuse large B-cell cutaneous lymphoma.

**Table 4:** Frequency of autoimmune diseases in patients with PCBCL and controls.

Comorbidities	PCBCL		Controls		*p* Value
Autoimmune diseases	number	%	number	%	
Autoimmune thyroiditis	9	16.1	5	21.7	
SLE	1	1.8	0	0	
Behcet’s syndrome	1	1.8	0	0	
Total	11	19.7	5	9.3	0.1764

Abbreviations: PCBCL, primary cutaneous B-cell lymphoma; SLE, systemic lupus erythematosus.

**Table 5:** Comparison of other comorbidities in patients with PCBCL and controls.

Comorbidities	PCBCL		Controls		*p* Value
Other	number	%	number	%	
Gastroenteritis	4	7.1	5	9.3	
GERD	3	5.4	6	11.1	
Ulcerative colitis	1	1.8	0	0	
Total	8	14.3	11	20.4	0.4558
Heart transplant	1	1.8	0	0	
HIV	1	1.8	0	0	
Hypogammaglobulinemia	1	1.8	0	0	
Total	3	5.4	0	0	0.2434
HBV	1	1.8	0	0	
HCV	3	7.1	0	0	
Total	4	0	1	1.9	0.3638
Type 2 Diabetes	11	19.6	1	1.9	0.0041 *
Psoriasis	1	1.8	3	5.6	0.3590

* Statistically significant values are reported and marked with *. Abbreviations: GERD, gastroesophageal reflux disease; HBV, hepatitis-B virus infection; HCV, hepatitis-C virus infection; HIV, human immunodeficiency virus infection; PCBCL, primary cutaneous B-cell lymphoma.

## Data Availability

Not applicable.
